# A network analysis of ICD-11 complex posttraumatic stress disorder symptoms in Danish treatment-seeking military veterans

**DOI:** 10.1186/s12888-026-07954-w

**Published:** 2026-03-14

**Authors:** Caroline Lund, Karen-Inge Karstoft, Anni B. S. Nielsen, Sofie Folke

**Affiliations:** 1 Research and Knowledge Centre, The Danish Veterans Centre, Danish Defence, Ryvangs Allé 1-3, Ringsted, Copenhagen, 2100 Denmark; 2 Department of Psychology, University of Copenhagen, Copenhagen, Denmark; 3 The Research Unit and Section of General Practice, Department of Public Health, University of Copenhagen, Copenhagen, Denmark; 4 Department of Military Psychology, The Danish Veterans Centre, Copenhagen, Denmark

**Keywords:** Complex PTSD, Network analysis, Military veterans, Central symptoms, ICD-11

## Abstract

**Background:**

Network analysis has been extensively used to study posttraumatic stress disorder (PTSD), but only a few studies have examined the network structure of ICD-11 complex PTSD (CPTSD). Currently, no networks of all CPTSD symptoms have been estimated on military samples. Therefore, this study aims to (1) explore the connections of CPTSD symptoms and (2) identify the most central symptoms in a clinical sample of military veterans presenting with CPTSD symptomatology.

**Method:**

Danish treatment-seeking soldiers and veterans completed the International Trauma Questionnaire prior to commencing treatment at the Military Psychology Department in the Danish Defense. Network psychometrics were used to analyze the symptom structure and centrality strength index of self-reported CPTSD symptoms in veterans meeting criteria for CPTSD (*N* = 279). Stability of our results was assessed following the recommended guidelines to ensure reliability.

**Results:**

Consistent with prior research, symptoms within the same CPTSD cluster were most strongly connected, except for affective dysregulation. Across all CPTSD symptoms, ‘external avoidance’ and ‘feelings of worthlessness’ emerged as the most central.

**Conclusion:**

These findings are consistent with prior networks reported in non-military samples (e.g., community samples) and may support the generalizability of the CPTSD symptom network across trauma-exposed groups.

**Clinical trial number:**

Not applicable.

**Supplementary Information:**

The online version contains supplementary material available at 10.1186/s12888-026-07954-w.

## Introduction

### Clinical impact statement

This study uses network analysis to investigate symptoms of complex posttraumatic stress disorder (CPTSD) in a sample of Danish treatment-seeking military veterans. CPTSD has shown to be more common among veterans than PTSD. However, the clinical importance of specific CPTSD symptoms is debated, as is their prioritization in treatment. In this study, feelings of worthlessness and trauma avoidance emerged as the most central symptoms.

As central symptoms are thought to play a role in symptom maintenance, negative self-appraisals and avoidance may represent relevant targets for future research. However, these findings are exploratory and require validation in prospective treatment studies before any clinical implications can be established.

Complex posttraumatic stress disorder (CPTSD) is recognized in the 11th revision of the International Classification of Diseases (ICD-11) as a new trauma-induced disorder next to posttraumatic stress disorder (PTSD) [1]. This inclusion reflects a debate among clinicians and trauma researchers about the ability of PTSD to adequately capture the complex set of symptoms that often arise from prolonged, repeated, or relational traumatization [2, 3]. Beyond classic PTSD symptoms of re-experiencing, avoidance, and sense of current threat, CPTSD is characterized by symptoms belonging to three clusters of (1) affective dysregulation (i.e., hyperactivation or emotional numbing), (2) interpersonal difficulties (i.e., feeling distant or emotionally cut-off from others), and (3) negative self-concept (i.e., feelings of worthlessness or being a failure) which collectively are referred to as *disturbances in self-organization* (DSO) [2, 4]. Despite the close relation between PTSD and CPTSD, PTSD is theoretically viewed as a fear response with the re-experiencing of the traumatic event and subsequent avoidance and hyperarousal as the driving mechanism behind this disorder [5]. While PTSD symptoms are directly related to the trauma, the additional DSO symptoms in CPTSD manifests across settings, independent of the trauma preceding them [6].

Accumulating evidence suggest that CPTSD is more prevalent than PTSD in clinical samples of military veterans [7–9]. A recent systematic review likewise found CPTSD to be more prevalent that PTSD in military and veteran populations [10]. While still not well-documented, some studies suggest that this might be related to prolonged combat exposure during deployment [8, 9], and the number of adverse childhood events documented in military populations [7, 9, 11]. Further, some evidence points to CPTSD being associated with greater functional impairment and more comorbidity in terms of anxiety and depression compared to PTSD [8, 12]. As such, research on whether CPTSD requires different treatment approaches than PTSD is needed [13]. Some studies show that conventional PTSD treatments effectively resolve CPTSD symptoms [14], whereas others find they are less efficient [15, 16]. In addition, one systematic review of psychological interventions for CPTSD demonstrated poorer outcomes for persons with CPTSD following childhood trauma [17]. As treatment outcomes for CPTSD are ambiguous and possibly less favorable for some groups, a more detailed exploration of CPTSD and its symptoms could be relevant for developing more tailored treatments.

Research on CPTSD has mostly applied latent variable models [18]. Following these models, symptoms reflect a latent variable (i.e. disorder) that acts as a common cause to the overt symptomatology, similar to symptoms in medical conditions [19, 20]. Even though most research in psychopathology adopt this view, there is a growing use of network models to study psychiatric disorders [21]. By comparison, the network approach defines mental disorders as a network of interconnected symptoms rather than a condition with a latent variable “causing” the symptoms [22]. The emergence and maintenance of disorders are described by the causal interaction between symptoms in a network initially started by the impact of an external factor [23]. As with PTSD, trauma exposure may initiate symptoms of re-experiencing that could give rise to avoidance of trauma-related stimuli, resulting in an ongoing sense of current threat [24]. Unlike latent variable models, network modeling makes it possible to explore the relation between symptoms and identify central symptoms that play a maintaining role for the disorder [25]. While debated in the psychopathology network literature, central symptoms have been suggested as potential treatment targets due to their influence on other symptoms in the network [23]. As such, studying the associations between PTSD and DSO symptoms in a network structure might be important on the path towards the development of effective full CPTSD-specific treatment approaches.

Compared to the amount of PTSD network analysis studies [26], only a few network studies have yet explored CPTSD symptoms [27–32] and only one in treatment-seeking military veterans [33]. Across these networks, there is a consistent pattern of the interconnectedness between CPTSD symptoms. ‘Feelings of worthlessness’ in the DSO cluster is the most reported central symptom followed by PTSD symptoms of avoidance. Differently, Hendrikx & Murphy (2023) identified affective dysregulation as the most central symptom cluster among treatment-seeking veterans. Being the only current network analysis of CPTSD conducted in a military population, these authors suggest that difficulties with affect regulation is characteristic for this group [33]. However, as this study investigated the association between clusters of CPTSD and not individual symptoms [33], it is not possible to make inferences at a symptom level [34]. Furthermore, there is evidence suggesting that symptoms of affect dysregulation in the CPTSD diagnosis reflect two opposing emotion regulation strategies [29–31]. Estimating networks at a symptom level, however, would provide insight into possible symptom variations.

In the current study, we aim to explore the network structure of all symptoms of ICD-11 CPTSD in a clinical sample of Danish treatment-seeking military soldiers and veterans. Based on prior research, we outline and test two hypotheses. Firstly, stronger connections are expected within the same ICD-11 CPTSD cluster, except for a weak association between affect dysregulation symptoms, as a result of their potential opposing nature. Secondly, as found in previous studies, ‘feelings of worthlessness’ is hypothesized to have the highest strength centrality [27–30, 35].

## Methods

### Sample

The study included previously deployed Danish military soldiers and veterans (both labeled ‘veterans’) seeking treatment at the Military Psychology Department (MPD), within the Danish Veterans Centre, Danish Defense, between 26th October 2019 and 16th October 2021 (*N* = 608). Participants were consecutively recruited from this national clinical service for treatment-seeking veterans. Data were retrieved from an online-distributed questionnaire required to be completed before treatment intake as part of a standardized procedure at the MPD. Because of this intake procedure, there were no missing data. Only data from the first referral were utilized if veterans had been referred more than once during the inclusion period. Responses were also screened for completeness and patterned responding (e.g., invariant or repetitive response pattern) to ensure data quality. For this study, eligible veterans were those that screened positive for at least one traumatic event on the Traumatic Life Events Questionnaire (TLEQ) [36] and met criteria for a probable diagnosis of CPTSD as assessed with the International Trauma Questionnaire (ITQ) [37], resulting in a subsample of *N* = 279. Thus, 329 participants were excluded due to not meeting criteria for probable CPTSD and/or not reporting at least one traumatic event on the TLEQ. Sample characteristics are provided in Table 1. As can be seen, the mean age was 42.3 years (SD = 9.10, Range: 23–67 years), and the majority were men (93.9%).

**Table 1:** Sample characteristics

**Sample size**	279
Age mean (SD)	42.3 (9.1)
Men, n (%)	262 (93.9)
Children (yes, n (%))	205 (73.5)
**Marital status**,** n (%)**	
Single	55 (19.7)
Girlfriend/Boyfriend (living separately)	32 (11.5)
Married (living together)	149 (53.4)
Divorced/Separated	41 (14.7)
Widow	2 (0.7)
**Employment status**,** n (%)**	
On sick leave	38 (13.6)
Student	18 (6.5)
Employed	129 (46.2)
Retired	35 (12.5)
Other	59 (21.1)
**PTSD symptoms**,** mean (SD)**	
Distressing dreams (RE1)	2.7 (1.1)
Intrusive recollections (RE2)	2.8 (0.9)
Internal avoidance (AV1)	3.0 (0.8)
External avoidance (AV2)	3.0 (1.0)
Sense of current threat (TH1)	3.3 (0.9)
Exaggerated startle response (TH2)	3.1 (1.0)
**DSO symptoms**,** mean (SD)**	
Long-time upset (hyperactivation) (AD1)	3.0 (0.9)
Emotional numbing (deactivation) (AD2)	3.1 (1.0)
Feelings of failure (NSC1)	3.1 (0.9)
Feelings of worthlessness (NSC2)	3.0 (1.0)
Feeling distant or cut off from others (DR1)	3.3 (0.8)
Difficulties feeling close to others (DR2)	3.1 (0.9)
**PTSD clusters**,** mean (SD)**	
Re-experiencing (RE)	5.5 (1.7)
Avoidance (AV)	6.0 (1.5)
Sense of current threat (TH)	6.3 (1.6)
**DSO clusters**,** mean (SD)**	
Affective dysregulation (AD)	6.1 (1.5)
Negative self-concept (NSC)	6.1 (1.7)
Disturbances in relationships (DR)	6.5 (1.4)

Note. All veterans fulfilled criteria for probable CPTSD

### Measures

#### Socio-demographics

The online questionnaire included socio-demographic questions about age, gender (female/male), marital status (single, divorced, widowed, in a relationship, cohabitating or married), employment status (on sick leave, student, employed, retired or other) and children (yes/no).

#### Traumatic events

Trauma exposure was measured using the TLEQ to screen for traumatic life events [36]. The TLEQ lists 19 types of traumatic events that may have occurred throughout an individual’s life, such as combat trauma, or natural disaster. The score for each item represents the number of trauma exposures, with the maximum score of six indicating that the trauma has occurred six or more times.

### ICD-11 CPTSD

CPTSD symptoms were assessed with the validated ITQ self-report questionnaire [38]. The ITQ consist of 12 items that measure symptoms in clusters of PTSD and DSO (CPTSD): Re-experiencing (RE) – Distressing dreams (RE1) & Intrusive recollections/flashbacks (RE2), Avoidance (AV) – Internal avoidance (AV1) & External avoidance (AV2), Sense of current threat (TH) – Hypervigilance/sense of current threat (TH1) & Exaggerated startle response (TH2), Affective dysregulation (AD) – Long-time upset (hyperactivation) (AD1) & Emotional numbing (hypoactivation) (AD2), Negative self-concept (NSC) – Feelings of failure (NSC1) & Feelings of worthlessness (NSC2) and Disturbance of relationships (DR) – Feeling distant or cut off from others (DR1) & Difficulties feeling close to others (DR2). All items were rated on a 5-point Likert scale from 0 (‘Not at all’) to 4 (’Extremely’). A score of ≥ 2 (’Moderately’) indicates the presence of a symptom that must also cause social or occupational impairment. As the ITQ is a self-report measure, (C)PTSD caseness is considered a probable diagnosis rather than a clinical confirmed diagnosis. For this study, a Danish version of the ITQ was utilized [39]. Cronbach’s Alpha in this study indicated good internal reliability for the total scale (α = 0.801), and acceptable reliability for the two subscales of PTSD (α = 0.726) and the DSO (α = 0.725).

### Statistical analyses

All statistical analyses were conducted in *R* version 4.2.3 in October 2023. By following standard statistical practice for network analysis, three steps were taken: (1) Network estimation, (2) Network description and (3) Network stability analyses [40–42]. For transparent reporting, we adhere to the reporting standards for psychological network analysis [34]. As the present study used a regularized network model rather than a regression model, assumptions such as normality, linearity, homoscedasticity, and multicollinearity were not formally tested, as they are not directly applicable to this approach. Model accuracy and stability were instead evaluated using recommended network-specific diagnostics (see below). Notable, there was not an a priori sample size calculation for this study. The code for the statistical analyses is available online. In network psychometrics, symptoms are referred to as *nodes* and the connection between nodes as *edges* [20]. Therefore, the network in this study included 12 nodes as assessed with the ITQ. Symptoms are referred to with abbreviations in the results and discussion section. See Table 1 for full symptom names.

#### Network estimation

The CPTSD network was estimated and visualized using *Gaussian graphical models* (GGM) for ordinal data via the R-package *qgraph* version 1.9.4 [43]. GGM represents partial correlations between nodes that remain after adjusting for all other nodes in the network. The *EBICglasso* algorithm was selected, specifically the *glasso* [44] based on the Extended Bayesian Information Criterion (EBIC) [45]. The *EBICglasso* is a regularization procedure that eliminates spurious associations by shrinking weak connections to zero. The default value in the EBIC computation was used with a gamma hyperparameter (γ) set to 0.50. For visualization, the Fruchterman–Reingold algorithm (labelled *spring*) was applied to place strongly connected nodes closer together in the network [46]. A color-blind theme in *qgraph* was additionally chosen [43].

#### Network description

Node centrality was estimated via the R-package *qgraph* [43] and used to identify nodes of higher importance in the network. Specifically, centrality strength [47] was assessed to explore each node’s influence based on its direct connections to other nodes in the network. Other common centrality metrics such as closeness or betweenness were not estimated, since they have proven to be unstable measures of the node importance in psychological networks and likewise challenging to interpret [48].

#### Network stability analyses

**Fig. 1:**
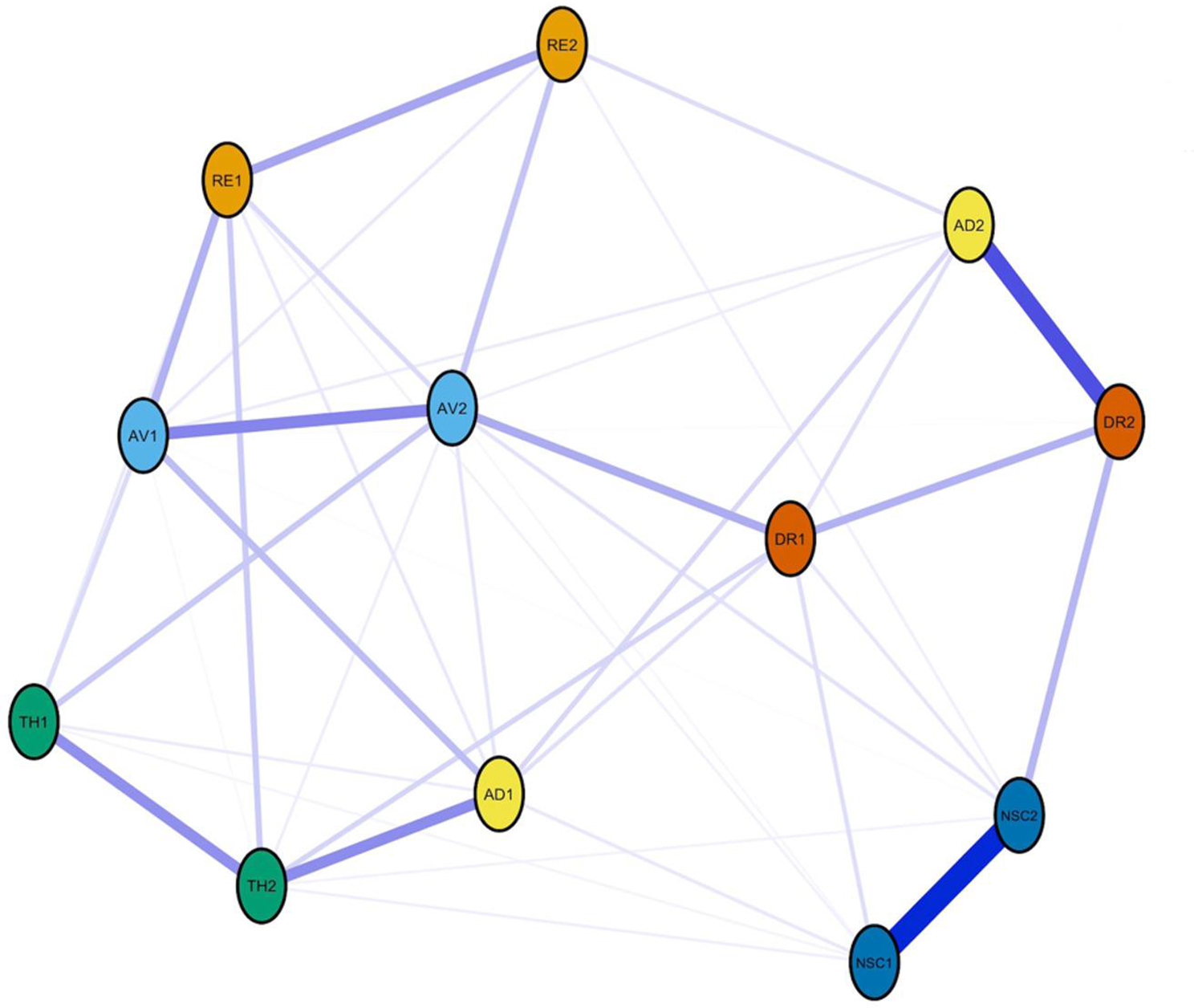
The Regularized Partial Correlation Network of CPTSD Symptoms in a Sample of Danish Treatment-Seeking Military Veterans. Note: A thick edge between nodes indicates stronger regularized partial correlation. Blue edges represent positive connections. Abbreviations: RE1 = Distressing dreams, RE2 = Intrusive recollections/flashbacks, AV1 = Internal avoidance, AV2 = External avoidance, TH1 = Hypervigilance/Sense of current threat, TH2 = Exaggerated startle response, AD1 = Long-time upset (hyperactivation), AD2 = Emotional numbing (hypoactivation), NSC1 = Feelings of failure, NSC2 = Feelings of worthlessness, DR1 = Feeling distant or cut off from others and DR2 = Difficulties feeling close to others

**Fig. 2:**
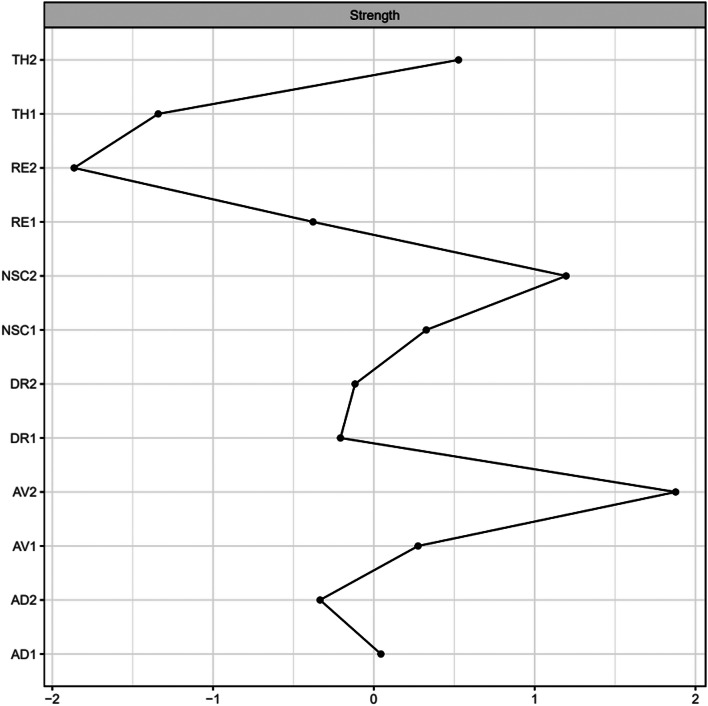
Centrality Strength of CPTSD Symptoms Converted to Z-Scores for Interpretation. Note: Abbreviations: RE1 = Distressing dreams, RE2 = Intrusive recollections/flashbacks, AV1 = Internal avoidance, AV2 = External avoidance, TH1 = Hypervigilance/Sense of current threat, TH2 = Exaggerated startle response, AD1 = Long-time upset (hyperactivation), AD2 = Emotional numbing (hypoactivation), NSC1 = Feelings of failure, NSC2 = Feelings of worthlessness, DR1 = Feeling distant or cut off from others and DR2 = Difficulties feeling close to others

To assess accuracy of weight edges, we used non-parametric bootstrapping with a 1,000 bootstrap samples implemented in the R-package *bootnet* version 1.5.3 [41]. Figure 1S in the online supplementary material depicts the 95% confidence intervals (CIs) around the edges. Additionally, we checked the stability of centrality strength estimates using case-drop bootstrapping via the *bootnet R-*package. The stability plot of centrality strength, generated with the subset bootstrapping technique, is illustrated in Fig. 3S in the online supplementary material. Bootstrapped difference-tests of both weight edges (Fig. 2S in the online supplementary material) and centrality (Fig. 4S in the online supplementary material) were also conducted to explore if some edges or centrality nodes within the network differed significantly from each other. Lastly, correlation-stability coefficients (CS-coefficient) were computed for weight edges and centrality strength, ranging from 0 to 1. Statistically, a CS-coefficient > 0.50 is recommended and not below the threshold of 0.25 [41]. Accordingly, CS values below this threshold should be interpreted with caution, as centrality estimates may be unstable and sensitive to sample differences. All models generated from the stability analyses are available in the online supplementary material.

## Results

### Network stability analyses

The bootstrapped confidence intervals (CIs) around the estimated edge weights showed consistency with the bootstrap estimates (Fig. 1S in the online supplementary material). According to centrality strength, the stability analyses indicated that the CS-coefficient exceeded the accepted stability level under subsetting cases (CS = 0.28) [40]. As such, this estimate must be interpreted as indicative, not conclusive.

### Network of CPTSD symptoms

Figure 1 visualizes the estimated partial correlation network of the 12 CPTSD symptoms. A high density was observed, with 43 edges retained out of possible 66 edges in the glasso network estimation, accounting for 65% of all possible edges. The mean edge weight was 0.07, and all symptoms were positively correlated. The strongest edges were identified between NSC1-NSC2 (regularized partial correlation (RPC) = 0.58), AD2-DR2 (RPC = 0.40) and AV1-AV2 (RPC = 0.28). Of note, the significance test of edge differences indicated that these edges differed significantly from other connections in the network (Fig. 2S in the online supplementary material). Otherwise, many edge weights did not differ significantly from each other. All numerical RPCs can be viewed in Table 1S in the online supplementary material.

### Centrality strength

Figure 2 depicts the standardized centrality estimates of strength for every node in the CPTSD network. The three symptoms with the highest centrality were AV2, NSC2 and TH2. Based on the bootstrapped difference-tests, AV2 and NSC2 did not differ significantly from each other, indicating that they are of equal importance (Fig. 4S in the online supplementary material). RE2 was identified as the weakest node and may be denoted as a symptom of less importance in this network.

### Hypothesis tests

H1 (stronger within-cluster connections, except AD1-AD2) was partially supported. H2 (worthlessness having the highest strength) was supported; external avoidance demonstrated comparable centrality.

## Discussion

The current study represents the first network analysis of individual ICD-11 CPTSD symptoms conducted in a military sample. Our two pre-study hypotheses were partly and fully supported, respectively. First, we found stronger connections within the same ICD-11 CPTSD cluster, except for symptoms of affect dysregulation. Second, ‘feelings of worthlessness’ showed the highest strength centrality, along with ‘external avoidance’. Regarding the first hypothesis, the network displayed strong edges between symptoms within ICD-11 PTSD and DSO symptom clusters: RE1-RE2, AV1-AV2, TH1-TH2 and NSC1-NSC2 (see Fig. 1). Symptoms in the DSO cluster of interpersonal difficulties (DR1-DR2) were connected, however, unexpectedly, the stability analyses showed that this edge did not significantly differ from many other edges (Fig. 2S in the online supplementary material). Instead, DR1 was strongly connected to AV2, whereas DR2 showed a strong edge to AD2. The strong associations between the two latter symptoms (DR2-AD2) in our study are consistent with findings in PTSD networks based on DSM-5 symptoms [49, 50]. In general, AD2 reflects an emotional strategy to cope with emotions by detaching oneself from feelings instead of engaging with them [51]. Being detached from one’s feelings may lead to emotional disengagement from others, which could foster ‘difficulties feeling close to others’ (DR2).

As further hypothesized, this network showed a weak edge between symptoms of affect dysregulation (AD1-AD2), supporting previous research [27–32, 35]. Also noted in studies using latent variable models of CPTSD [52, 53], it can be questioned whether these two symptoms qualify to represent a unidimensional cluster of CPTSD. In our network, AD1 was more associated with PTSD symptoms, whereas AD2 was connected to DSO symptoms. Previous research supports our findings by showing that AD1 is more associated with PTSD and could act as an important bridge symptom between PTSD and DSO symptoms [28]. The divergence between AD1 and AD2 aligns with a conceptualization of two opposing emotion-regulation strategies. As suggested in previous studies [29–31], this might be reflected in an affect dysregulation cluster with limited coherence.

Overall, this network featured a cluster division of PTSD and DSO comparable to other network studies and corresponding to the ICD-11 classification of CPTSD [27–30, 35]. According to the network theory, strong symptom connections suggest a dynamic relationship and potential shared etiology [24]. Thus, feelings of failure and worthlessness (NSC1-NSC2) may be strongly connected due to a shared psychological thought mechanism. Activation of one of these symptoms may lead to a feedback loop of negative self-appraisal. However, as the network theory and its validity is under investigation [54], it is suggested using the network framework for more exploratory purposes [55]. This involves identifying robust empirical phenomena that may serve as precursors to a subsequent theory formation [55].

Support for the second hypothesis was also found since NSC2 is highly central (Fig. 2); adding to the findings of previous research [27–30, 35]. AV2 appeared to be the most central symptom in this network, however the stability analysis showed that this symptom did not significantly differ from NSC2 (Fig. 4S in the online supplementary material), pointing towards their potential equal importance. Notably, exaggerated startle (TH2) also showed relatively high centrality, consistent with the prominence of hyperarousal symptoms in veteran PTSD networks [49, 50]. However, as the CS-coefficient showed moderate stability (Cor=0.28), just above the threshold of 0.25, these estimates should be interpreted cautiously. Despite a lower degree of stability, the results of this study indicate that avoidance of trauma-related stimuli and feelings of worthlessness are central in the CPTSD network and, thereby, to treatment-seeking military veterans’ experience of traumatization. The centrality of avoidance in our study suggests that the fear response underlying PTSD may still be central to the CPTSD network, in addition to feelings of worthlessness included in the new CPTSD diagnosis.

Studies show that negative self-evaluations are a vulnerability factor for developing PTSD [56] and a cognitive maintenance factor [57]. Furthermore, a negative self-concept has been found to predict non-remittance in PTSD [58]. Concerning trauma-related avoidance, this is often regarded as a strategy to cope with distressing memories, feelings or thoughts associated with the trauma. Avoidance is also associated with PTSD severity and poorer treatment outcomes [59]. Thus, these studies provide support for the importance of NSC2 and AV2 [56, 57, 59].

Given that feelings of worthlessness, unlike avoidance symptoms, are associated with the additional DSO cluster specific to CPTSD, future research should explore whether feelings of worthlessness is more central in CPTSD than in PTSD. According to Ehlers & Clark’s cognitive model of PTSD (2000), a negative self-view, such as worthlessness, often follows trauma, where individuals experience a sense of mental defeat and a loss of psychological autonomy. Research has shown that feelings of mental defeat are associated with interpersonal traumas [60], characterizing CPTSD compared to PTSD [61]. In the context of our study, feelings of worthlessness among military veterans are not surprising, given the risk of exposure to multiple interpersonal traumas during deployment to war and conflict zones [62]. Since veterans, in general, respond less to traditional trauma-focused interventions for PTSD than non-veterans [15], results from our study could indicate research into new interventions for this group that target a negative-self view and avoidance and willingness to revisit the trauma. Nevertheless, it is essential to be cautious when interpreting centrality estimates [63, 64]. The network approach in psychopathological research is relatively new, and results from these studies are exploratory [65]. Accordingly, the identified central symptoms should be considered hypothesis-generating findings rather than predictive markers or definitive treatment targets.

### Limitations

There are some limitations of this study. First, this network is estimated on cross-sectional data. As such, edges are undirected. Future studies should use time-series data to investigate the directedness of symptom relations. Such studies could also show if the central symptom in current cross-sectional networks are a consequence of the other symptoms rather than their cause [66]. Second, the study was conducted with a highly homogeneous, exclusively male sample, which limits the extent to which these findings can be generalized to broader CPTSD populations. Demographic moderation analyses were not possible due to sample size constraints; therefore, the effects of gender and other socio-demographic factors represent an important avenue for future research. Third, findings are based on self-reported CPTSD symptoms assessed using the ITQ, which may be subject to response bias. Moreover, reliance on a single self-report method raises the possibility of common method variance, potentially inflating associations among symptoms and affecting the estimated network structure. Clinical interviews assessing CPTSD [67] could have provided more reliable CPTSD estimates. Diagnostic status should therefore be interpreted as probable; future work using clinician-administered interviews is warranted. Moreover, the online administration format may introduce unobservable response biases. Fourth, the sample size was small, which could explain the low stability of centrality results, the larger confidence intervals around edge weights, and may also influence network density. Regularization procedures were used in this study but are conservative when applied to small samples [41], highlighting the exploratory nature of the findings and the need for cautious interpretation. Finally, some edges were identified between symptoms following the item order in the ITQ. This could show an ‘order effect’ that refers to a phenomenon where a response to a prior item affects the response to the following item [68]. A way to reduce this risk of bias would be to shuffle the order of the items. Despite aforementioned limitations, the centrality symptoms identified in this study support previous research, showing that feelings of worthlessness are central to the CPTSD symptom constellation in various trauma exposed samples [27–30, 35], in addition to avoidance [29, 30, 35].

## Conclusion

Network studies of ICD-11 CPTSD symptoms are still limited. This is the first network analysis that has investigated the network structure of CPTSD symptoms in a clinical sample of military veterans. Strong connections were primarily found within CPTSD symptom clusters, and ‘feelings of worthlessness’ and ‘external avoidance’ were the most central symptoms. These results align with existing evidence conducted in other countries with different trauma exposed samples [27–30, 35]. Whereas some researchers suggest that the type of trauma exposure lead to different central CPTSD symptoms [32], these findings indicate that the network structure of CPTSD could be a robust finding across trauma groups, including treatment-seeking veterans. Now more research is needed, using different data collection methods, to confirm the generalizability of this network and explore the impact of various factors, such as the type of trauma, amount of exposure or cultural differences on the network structure of ICD-11 CPTSD.

## Supplementary Information

Below is the link to the electronic supplementary material.

Supplementary Material 1

Supplementary Material 2

Supplementary Material 3

## Data Availability

Due to the privacy and data protection regulations from the Danish Defence, the data are not publicly available. This study was not preregistered.
