# Supplementary material for: A network analysis of ICD-11 complex posttraumatic stress disorder symptoms in Danish treatment-seeking military veterans
Source: BMC Psychiatry. 2026 Mar 14;26:337. doi: 10.1186/s12888-026-07954-w (PMC13101302; doi:10.1186/s12888-026-07954-w)
Supplement: Supplementary file 1 — Supplementary Material 1 [file 12888_2026_7954_MOESM1_ESM.docx]

# **Supplementary material**

**“A Network Analysis of ICD-11 Complex Posttraumatic Stress Disorder Symptoms in Danish Treatment-Seeking Military Veterans”**

**Table 1S.** *Regularized partial correlations between ICD-11 CPTSD symptoms.*

|  | RE1 | RE2 | AV1 | AV2 | TH1 | TH2 | AD1 | AD2 | NSC1 | NSC2 | DR1 | DR2 |
| --- | --- | --- | --- | --- | --- | --- | --- | --- | --- | --- | --- | --- |
| RE1 | 0.000 | 0.205 | 0.174 | 0.085 | 0.049 | 0.117 | 0.050 | 0.000 | 0.029 | 0.000 | 0.000 | 0.000 |
| RE2 | 0.205 | 0.000 | 0.051 | 0.134 | 0.000 | 0.000 | 0.000 | 0.076 | 0.000 | 0.035 | 0.000 | 0.00 |
| AV1 | 0.174 | 0.051 | 0.000 | 0.278 | 0.082 | 0.013 | 0.150 | 0.043 | 0.000 | 0.004 | 0.000 | 0.004 |
| AV2 | 0.085 | 0.134 | 0.278 | 0.000 | 0.124 | 0.037 | 0.058 | 0.035 | 0.019 | 0.063 | 0.190 | 0.002 |
| TH1 | 0.049 | 0.000 | 0.082 | 0.124 | 0.000 | 0.253 | 0.042 | 0.000 | 0.023 | 0.000 | 0.000 | 0.000 |
| TH2 | 0.117 | 0.000 | 0.013 | 0.037 | 0.253 | 0.000 | 0.259 | 0.000 | 0.033 | 0.028 | 0.000 | 0.000 |
| AD1 | 0.050 | 0.000 | 0.150 | 0.058 | 0.042 | 0.259 | 0.000 | 0.088 | 0.050 | 0.000 | 0.095 | 0.000 |
| AD2 | 0.000 | 0.076 | 0.043 | 0.035 | 0.000 | 0.000 | 0.088 | 0.000 | 0.000 | 0.000 | 0.073 | 0.400 |
| NSC1 | 0.029 | 0.000 | 0.000 | 0.019 | 0.023 | 0.033 | 0.050 | 0.000 | 0.000 | 0.580 | 0.073 | 0.000 |
| NSC2 | 0.000 | 0.035 | 0.004 | 0.063 | 0.000 | 0.028 | 0.000 | 0.000 | 0.580 | 0.000 | 0.055 | 0.165 |
| DR1 | 0.000 | 0.000 | 0.000 | 0.190 | 0.000 | 0.095 | 0.070 | 0.073 | 0.073 | 0.055 | 0.000 | 0.176 |
| DR2 | 0.000 | 0.000 | 0.004 | 0.002 | 0.000 | 0.000 | 0.000 | 0.400 | 0.000 | 0.165 | 0.176 | 0.000 |

*Note.* Abbreviations: RE1 = Distressing dreams, RE2 = Intrusive recollections/flashbacks, AV1 = Internal avoidance, AV2= External avoidance, TH1 = Hypervigilance/Sense of current threat, TH2 = Exaggerated startle response, AD1 = Long-time upset (hyperactivation), AD2 = Emotional numbing (hypoactivation), NSC1 = Feelings of failure, NSC2 = Feelings of worthlessness, DR1 = Feeling distant or cut off from others and DR2 = Difficulties feeling close to others.

**Figure 1S. Accuracy analysis**. *95% bootstrapped confidence intervals (CIs) of edge weights in the network of 12 CPTSD symptoms. The grey area demonstrates the bootstrapped CIs, while the red line shows the edge weights of the sample. The y-axis is ordered with the lowest edge weights (bottom) to the highest edge-weights (top). A larger grey area indicates higher uncertainty or variability in the estimated edge weights, while a smaller grey area suggests more precise estimates.*


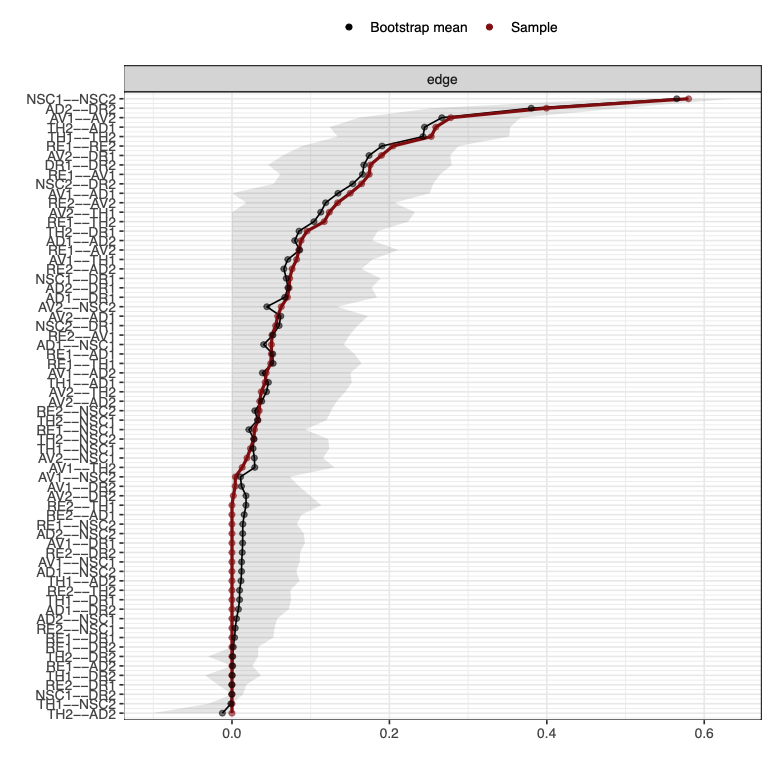


*Note.* Abbreviations: RE1 = Distressing dreams, RE2 = Intrusive recollections/flashbacks, AV1 = Internal avoidance, AV2= External avoidance, TH1 = Hypervigilance/Sense of current threat, TH2 = Exaggerated startle response, AD1 = Long-time upset (hyperactivation), AD2 = Emotional numbing (hypoactivation), NSC1 = Feelings of failure, NSC2 = Feelings of worthlessness, DR1 = Feeling distant or cut off from others and DR2 = Difficulties feeling close to others.

**Figure 2S. Edge weights difference test.** *A plot illustrating the results of bootstrapped significance test of the difference between the edge weights in the CPTSD network. Grey indicates no significant difference in edge weights, while black represents a significant difference. The diagonal boxes represent the edge weights.*


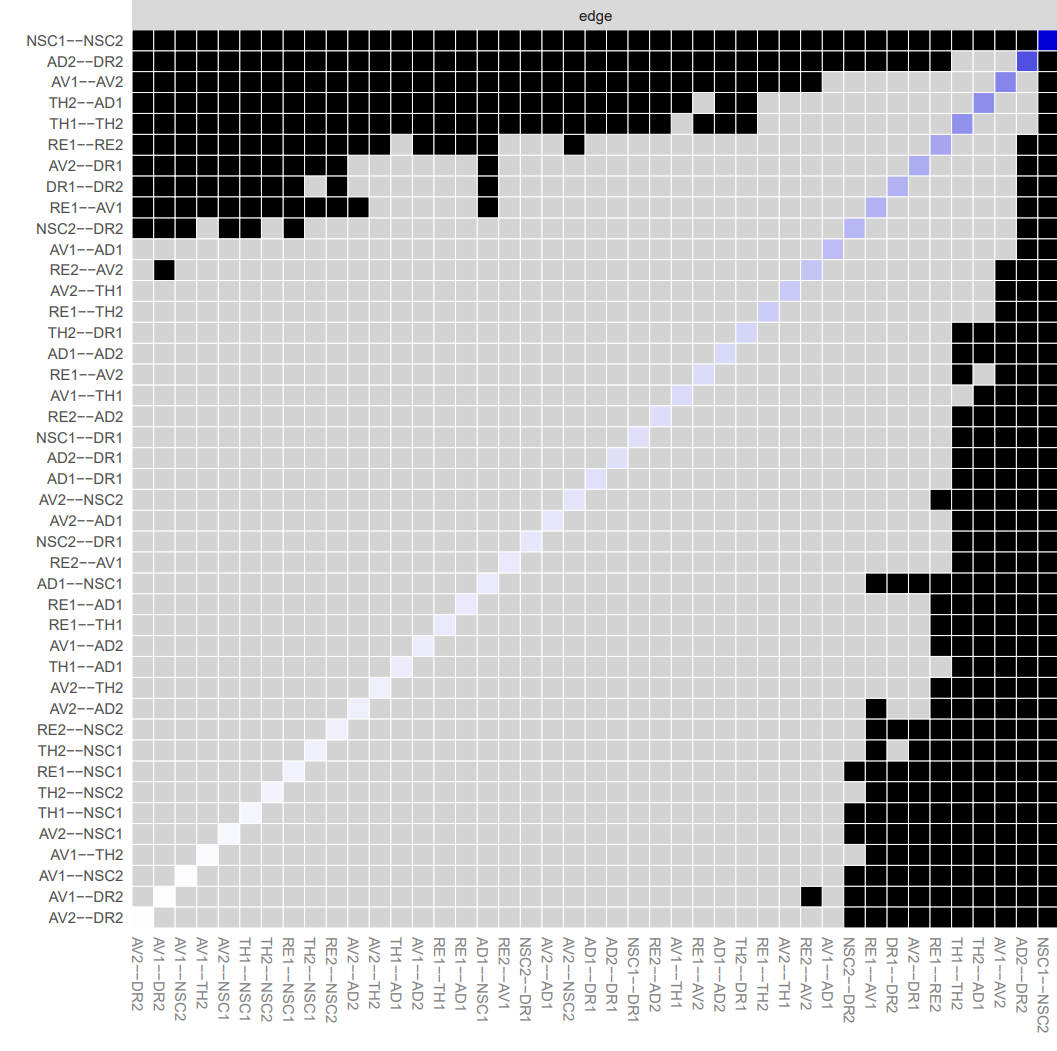


*Note.* Abbreviations: RE1 = Distressing dreams, RE2 = Intrusive recollections/flashbacks, AV1 = Internal avoidance, AV2= External avoidance, TH1 = Hypervigilance/Sense of current threat, TH2 = Exaggerated startle response, AD1 = Long-time upset (hyperactivation), AD2 = Emotional numbing (hypoactivation), NSC1 = Feelings of failure, NSC2 = Feelings of worthlessness, DR1 = Feeling distant or cut off from others and DR2 = Difficulties feeling close to others.

**Figure 3S.** *Stability plot of centrality strength conducted with the subset bootstrapping technique.*

*
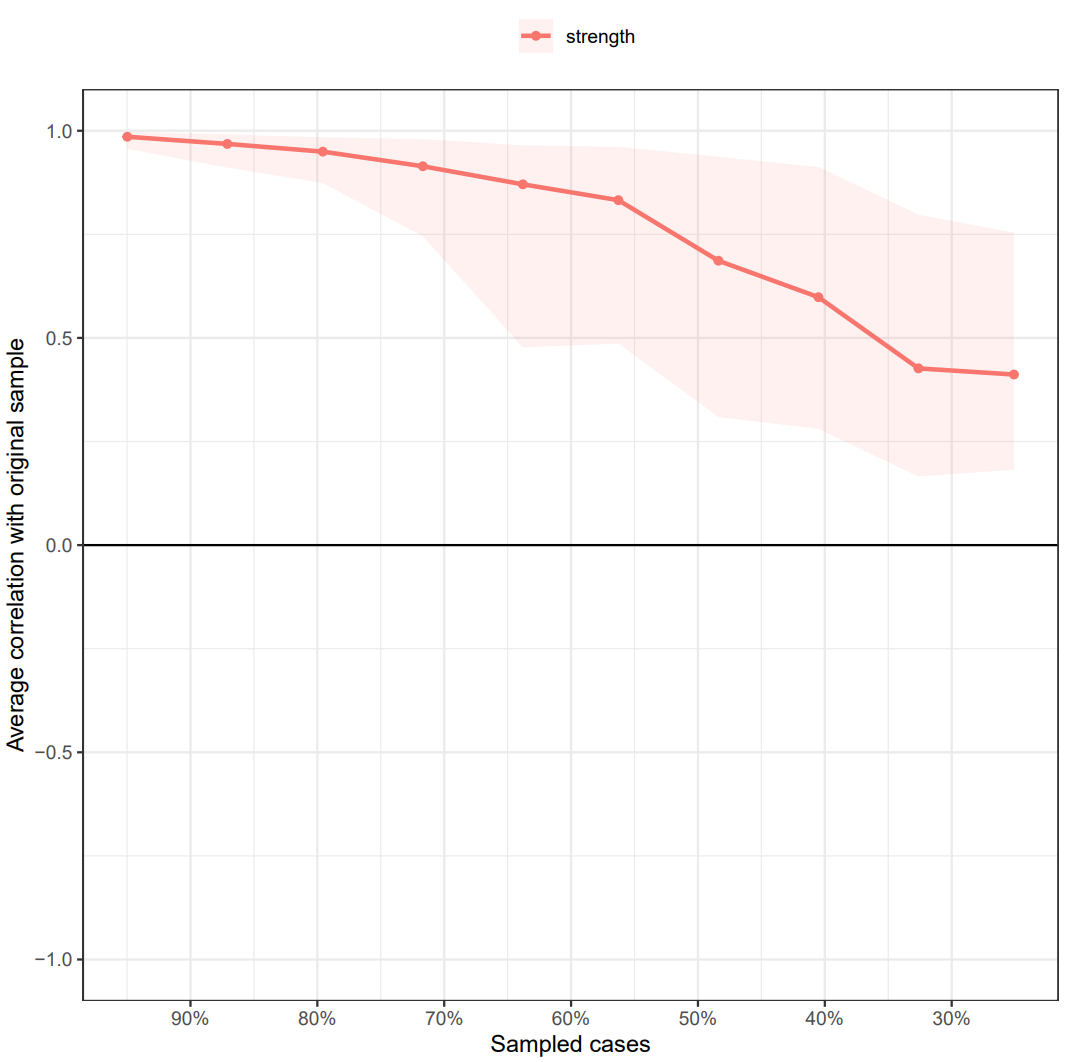
*

**Figure 4S.** *A bootstrapped difference test of centrality strength. A black box signifies that the node strength differs significantly from the other nodes, and the opposite is true for grey boxes. The diagonal values represent the value of node strength*.


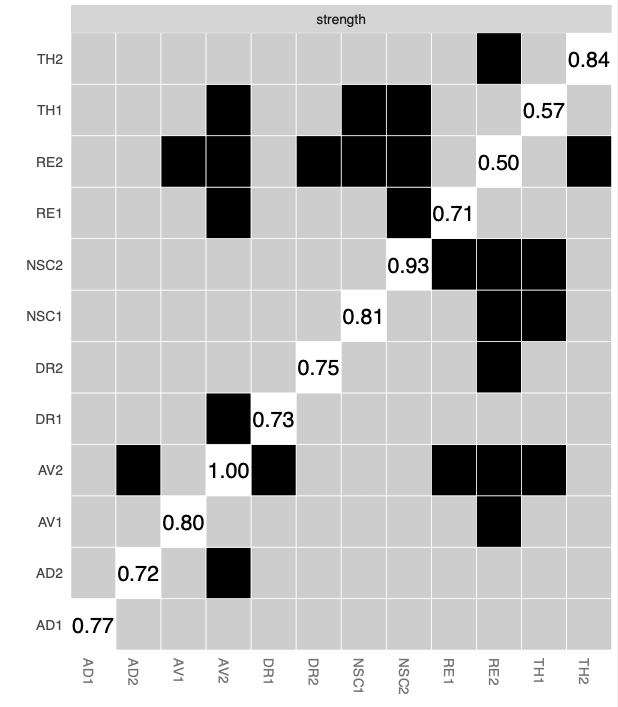


*Note*. Abbreviations: RE1 = Distressing dreams, RE2 = Intrusive recollections/flashbacks, AV1 = Internal avoidance, AV2= External avoidance, TH1 = Hypervigilance/Sense of current threat, TH2 = Exaggerated startle response, AD1 = Long-time upset (hyperactivation), AD2 = Emotional numbing (hypoactivation), NSC1 = Feelings of failure, NSC2 = Feelings of worthlessness, DR1 = Feeling distant or cut off from others and DR2 = Difficulties feeling close to others.
