# Supplementary material for: A network analysis of ICD-11 complex posttraumatic stress disorder symptoms in Danish treatment-seeking military veterans
Source: BMC Psychiatry. 2026 Mar 14;26:337. doi: 10.1186/s12888-026-07954-w (PMC13101302; doi:10.1186/s12888-026-07954-w)
Supplement: Supplementary file 3 — Supplementary Material 3 [file 12888_2026_7954_MOESM3_ESM.docx]

#Network analysis of ICD-11 CPTSD symptoms

#Load data

library(haven)

data <-read_sav("data")

View(data)

#Loading other libraries for later use

library("qgraph")

library("bootnet")

library("corpcor")

library("ggplot2")

#Rename variables and for this analysis CPTSD symptoms

names(data)[names(data) == "itq2_p1"] <- "RE1"

names(data)[names(data) == "itq2_p2"] <- "RE2"

names(data)[names(data) == "itq2_p3"] <- "AV1"

names(data)[names(data) == "itq2_p4"] <- "AV2"

names(data)[names(data) == "itq2_p5"] <- "TH1"

names(data)[names(data) == "itq2_p6"] <- "TH2"

names(data)[names(data) == "itq2_c1"] <- "AD1"

names(data)[names(data) == "itq2_c2"] <- "AD2"

names(data)[names(data) == "itq2_c3"] <- "NSC1"

names(data)[names(data) == "itq2_c4"] <- "NSC2"

names(data)[names(data) == "itq2_c5"] <- "DR1"

names(data)[names(data) == "itq2_c6"] <- "DR2"

names(data)

#Labels ITQ

legend <- c("Distressing dreams","Intrusive recollections/flashbacks",

"Internal avoidance", "External avoidance",

"Hypervigilance/Sense of current threat",

"Exaggerated startle response","Long-time upset (hyperactivation)",

"Emotional numbing (hypoactivation)",

"Feelings of failure", "Feelings of worthlessness",

"Feeling distant or cut off from others",

"Difficulities feeling close to others")

#Constructing a new dataset with only ITQ symptoms

Data1 <- data[c("RE1", "RE2", "AV1", "AV2", "TH1",

"TH2", "AD1", "AD2", "NSC1", "NSC2", "DR1", "DR2")]

#1 Sample Descriptives (See Table 1 in article)

library(summarytools)

view(dfSummary(data))

#vector - Employment_Status

Employment_Status <- vector("character", length = nrow(data))

#Descriptives for Employment status

for (i in 1:nrow(data)) {

if (data$q0005_0013[i] == 1) {

Employment_Status[i] <- "On Sick Leave"

} else if (data$q0005_0007[i] == 1 | data$q0005_0008[i] == 1) {

Employment_Status[i] <- "Student"

} else if (data$q0005_0009[i] == 1 | data$q0005_0011[i] == 1) {

Employment_Status[i] <- "Retired"

} else if (data$q0005_0001[i] == 1 | data$q0005_0003[i] == 1 | data$q0005_0004[i] == 1 | data$q0005_0005[i] == 1 | data$q0005_0006[i] == 1) {

Employment_Status[i] <- "Employed"

} else if (data$q0005_0002[i] == 1 | data$q0005_0012[i] == 1 | data$q0005_0016[i] == 1 | data$q0005_0010[i] == 1 | data$q0005_0014[i] == 1 | data$q0005_0015[i] == 1) {

Employment_Status[i] <- "Other"

} else {

Employment_Status[i] <- "Unknown"

}

}

#Overview - Employment status

view(dfSummary(Employment_Status))

#2 Estimation of the regularized partial correlation network of CPTSD symptoms

library(qgraph)

ggm <- estimateNetwork(Data1, default = "EBICglasso", verbose = FALSE)

#2.1 Extracting the adjacency matrix

adjacency_matrix_GGM <- getWmat(ggm)

View(adjacency_matrix_GGM)

#2.1.1 The same matrix as extracted above, however now with 3 decimals

View(round(adjacency_matrix_GGM, 3))

#2.2 Defining my groups

groups_list <- list(

RE = c("RE1", "RE2"),

AV = c("AV1", "AV2"),

TH = c("TH1", "TH2"),

AD = c("AD1", "AD2"),

NSC = c("NSC1", "NSC2"),

DR = c("DR1", "DR2")

)

#Colorblind-friendly for the groups

node_colors <- c(

"#E69F00", "#E69F00", # RE1, RE2

"#56B4E9", "#56B4E9", # AV1, AV2

"#009E73", "#009E73", # TH1, TH2

"#F0E442", "#F0E442", # AD1, AD2

"#0072B2", "#0072B2", # NSC1, NSC2

"#D55E00", "#D55E00" # DR1, DR2

)

#2.3 Plotting the regularized ggm

qgraph(adjacency_matrix_GGM)

#2.4# Making the ggm more fancy by using a colorblind-friendly theme and

#the layout spring

qgraph(adjacency_matrix_GGM,

layout = "spring",

color = node_colors,

legend = FALSE,

legend.cex = 0.4,

label.cex = 1.0,

vsize = 4,

border.width = 2.0,

theme = "colorblind",

GLratio = 1.4,

legend.cex = .35)

#Saving my ggm plot as a pfd

pdf("Regularized GGM CPTSD_2.pdf")

qgraph(adjacency_matrix_GGM,

layout = "spring",

color = node_colors,

legend = FALSE,

legend.cex = 0.4,

label.cex = 1.0,

vsize = 4,

border.width = 2.0,

theme = "colorblind",

GLratio = 1.4,

legend.cex = .35)

#3 Network description - centrality strenght

#Standardized centrality strenght

Strenght <- centralityPlot(adjency_matrix_GGM, scale = "z-scores")

#Saving the strenght plot as pdf

pdf("Standardized_strenght.pdf")

centralityPlot(adjency_matrix_GGM, scale = "z-scores")

dev.off()

#####Stability analysis for edge weight and centrality strenght########

#4.1 Checking accuracy of the estimated edge weights

#by using **nonparametric bootstrapping with replacement** to construct 95% bootstrapped confidence intervals.

#Setting seed for reproducibility

set.seed(1)

#Running the non-parametric bootstrap

boot.edges <- bootnet(ggm, boots = 1000, nCore = 8, verbose = FALSE)

#Plotting the 95% bootstrapped confidence intervals

plot(boot.edges, labels = TRUE, order = "sample")

#Saving the plot as a pfd

pdf("BOOTNET1.pdf")

plot(boot.edges, labels = TRUE, order = "sample")

dev.off()

#Edge weights difference plot

plot(boot.edges, "edge", plot = "difference", onlyNonZero = TRUE,

order = "sample")

#Saving the plot as a pdf

pdf("BOOTNET2_DIFFERENCETEST.pdf")

plot(boot.edges, "edge", plot = "difference", onlyNonZero = TRUE, order = "sample")

dev.off()

#4.2 Checking centrality stability

#by using the **case-dropping subset bootstrap**

#Setting seed for reproducibility

set.seed(2)

#Running the case-dropping subset bootstrap

boot.centrality <- bootnet(ggm, boots = 1000, nCores = 8, type = "case", verbose = FALSE)

#Centrality bootstrap plot

plot(boot.centrality)

#Save the plot as a pdf

pdf("BOOTNET3CENTRALITY.pdf")

plot(boot.centrality)

dev.off()

#Centrality difference plot for strength centrality

plot(boot.edges, "strength", plot = "difference")

#Save the plot as a pdf

pdf("BOOTNET4CENTRALITYDIFFERENCETEST.pdf")

plot(boot.edges, "strength", plot = "difference")

dev.off()

#Numerical results of the CS-coefficient

Correlation_koefficienter <- corStability(boot.centrality)

round(Correlation_koefficienter, 2)

View(Correlation_koefficienter)

#Done
